# Supplementary material for: Mapping disparities in viral infection rates using highly multiplexed serology
Source: mSphere. 2024 Aug 20;9(9):e00127-24. doi: 10.1128/msphere.00127-24 (PMC11423740; doi:10.1128/msphere.00127-24)
Supplement: Supplemental Figures — Figures S1 to S6. [file msphere.00127-24-s0001.pdf]

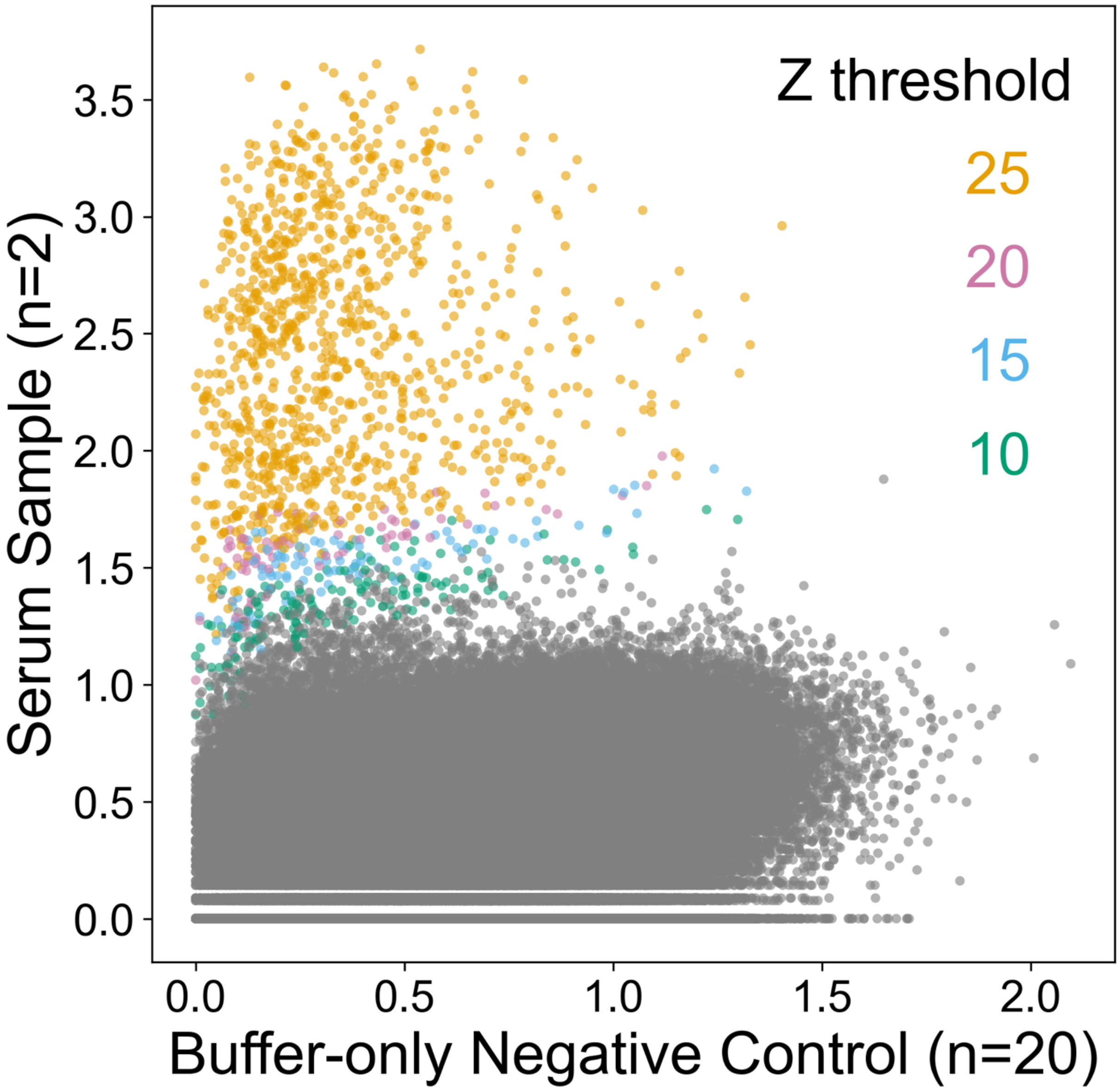

Supplemental Figure 1. Example of PepSeq results for a single serum sample highlighting peptides that have been enriched through antibody interactions. Scatterplot showing log-transformed estimates of relative abundance (reads per million) for a single serum sample run in duplicate (y-axis) compared to buffer only controls (x-axis, average of 20 replicates shown). Enriched peptides are colored according to the maximum Z score threshold cutoff at which they were considered enriched (as indicated in the upper right). Unenriched peptides are shown in gray.

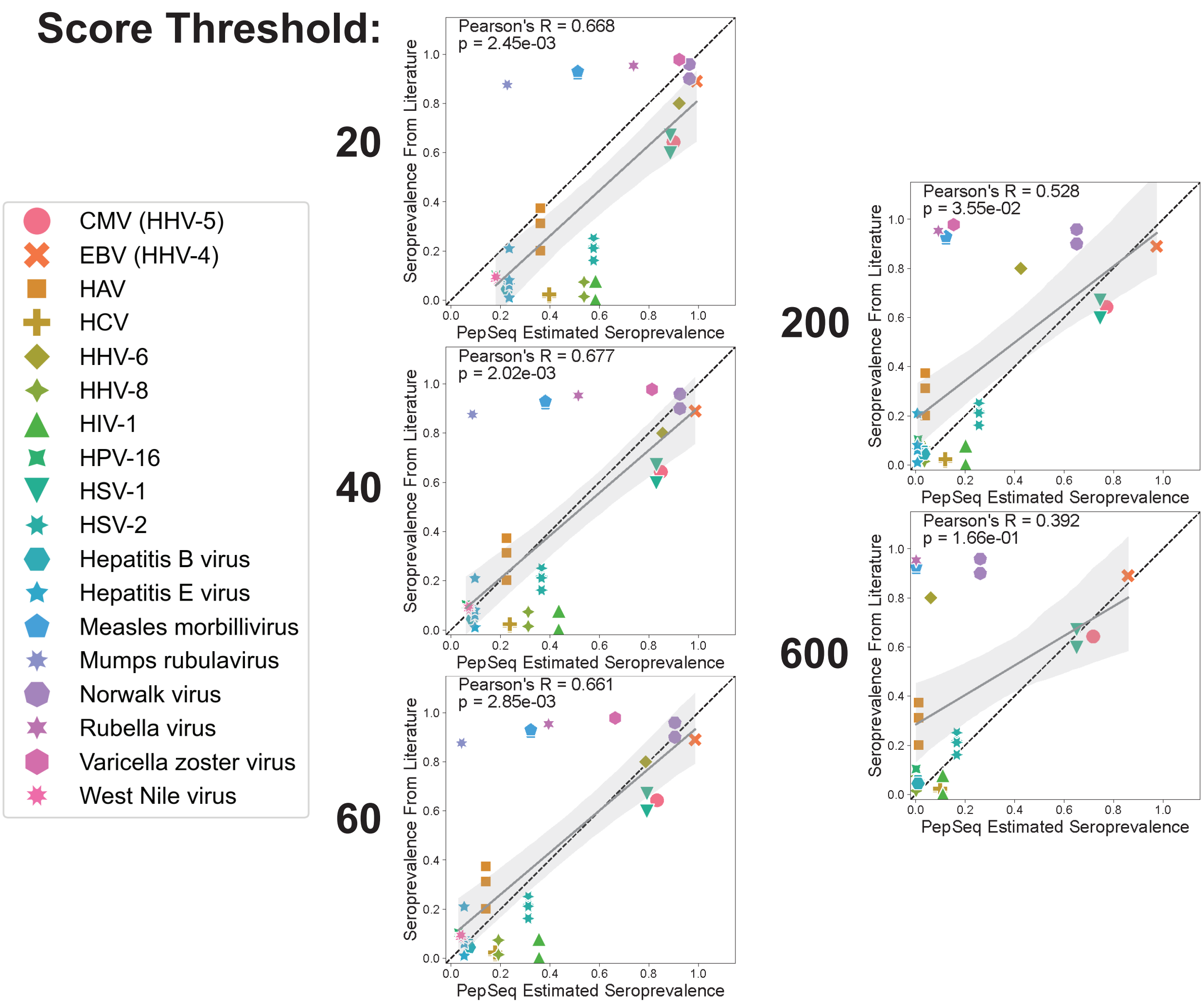

Supplemental Figure 2. Correlations between PepSeq estimated seroprevalence using fixed seropositivity score thresholds and seroprevalence estimates from published studies. PepSeq estimates of seroprevalence were generated using a Z score threshold of 15 and fixed seropositivity score thresholds of 20, 40, 60, 200 and 600 (as indicated to the left of each plot). The gray diagonal line indicates the best-fit linear regression with the shaded gray areas showing the 95% confidence interval. Dashed line indicates x=y. Pearson's R value and p-value shown in the top left of each plot. Supporting information for literature-derived estimates of seroprevalence can be found in Table S1. Abbreviations: CMV=cytomegalovirus, EBV=Epstein-Barr virus, HAV=hepatitis A virus, HCV=hepatitis C virus, HHV=human herpesvirus, HIV=human immunodeficiency virus, HPV=human papillomavirus, HSV=herpes simplex virus.

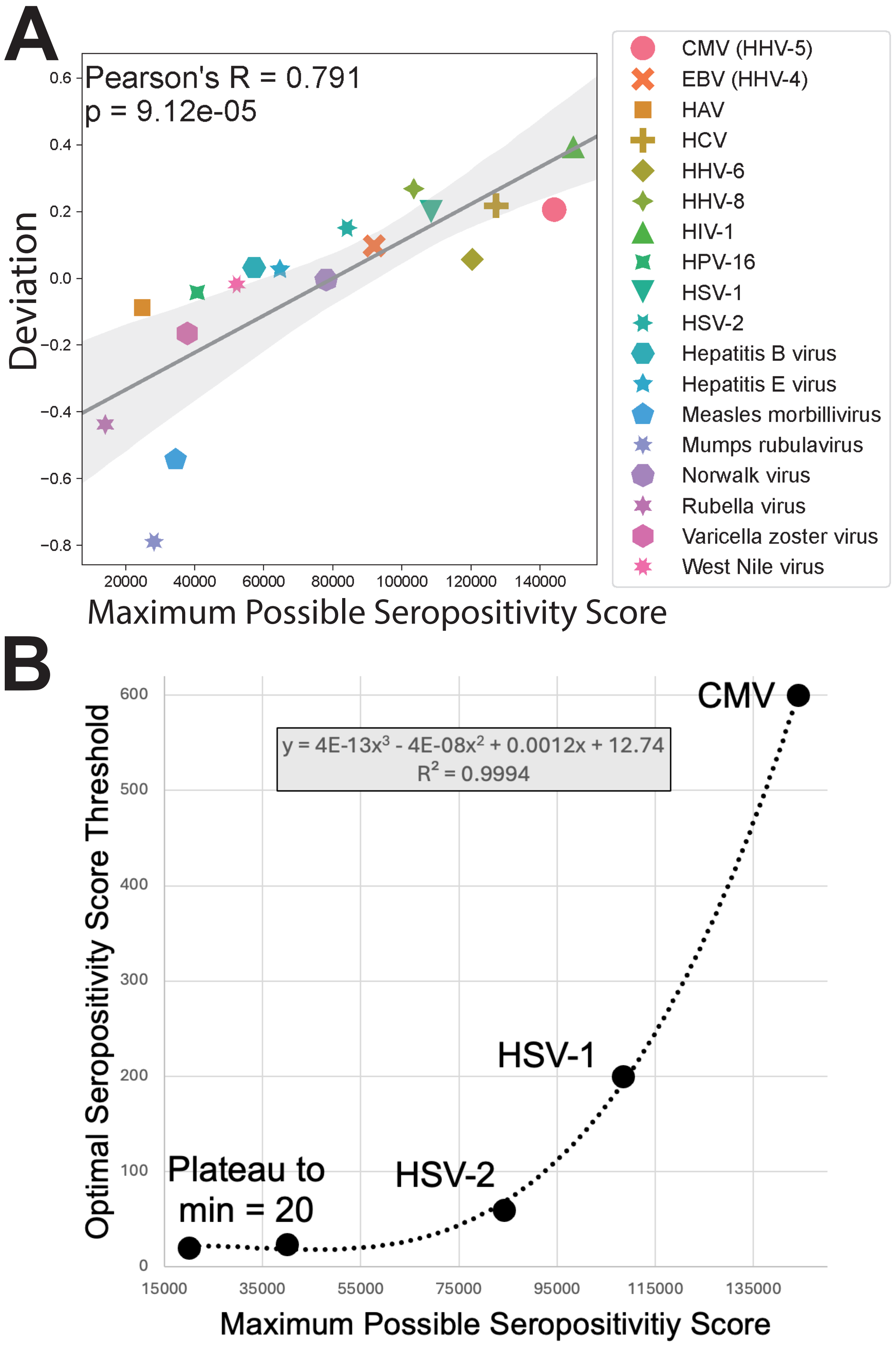

A
Pearson's R = 0.791
p = 9.12e-05
Deviation
0.6
0.4
0.2
0.0
−0.2
−0.4
−0.6
−0.8
20000
40000
60000
80000
100000
120000
140000
Maximum Possible Seropositivity Score
CMV (HHV-5)
EBV (HHV-4)
HAV
HCV
HHV-6
HHV-8
HIV-1
HPV-16
HSV-1
HSV-2
Hepatitis B virus
Hepatitis E virus
Measles morbillivirus
Mumps rubulavirus
Norwalk virus
Rubella virus
Varicella zoster virus
West Nile virus
B
y = 4E-13x³ - 4E-08x² + 0.0012x + 12.74
R² = 0.9994
CMV
HSV-1
HSV-2
Plateau to
min = 20
Optimal Seropositivity Score Threshold
600
500
400
300
200
100
0
15000
35000
55000
75000
95000
115000
135000
Maximum Possible Seropositivitiy Score

Supplemental Figure 3. Representation bias within our PepSeq library influences the optimal seropositivity score threshold for a virus. A) Scatterplot comparing the deviation in estimated seroprevalence between PepSeq and published studies using singleplex assays (y-axis) to the maximum possible seropositivity score in our HV1 PepSeq library (x-axis). Maximum possible seropositivity score provides an indication of how well represented a virus is within our HV1 PepSeq library. The gray diagonal line indicates the best-fit linear regression with the shaded gray areas showing the 95% confidence interval. Pearson's R value and p-value shown in the top left. B) Scatterplot comparing the maximum possible seropositivity score in the HV1 PepSeq library (x-axis) to the optimal seropositivity score thresholds calculated from the ELISA ROC curves (y-axis) shown in Figure 3C. Dotted line indicates a 3rd order polynomial line fit to the virus data by Excel, with the addition of two data points at x=20,000 and x=40,000 to ensure a plateau, on the left, at a seropositivity score threshold of 20. The equation of the fit line and it's R^2 value is shown in the grey box in the upper left. Abbreviations: CMV=cytomegalovirus, EBV=Epstein-Barr virus, HAV=hepatitis A virus, HCV=hepatitis C virus, HHV=human herpesvirus, HIV=human immunodeficiency virus, HPV=human papillomavirus, HSV=herpes simplex virus.

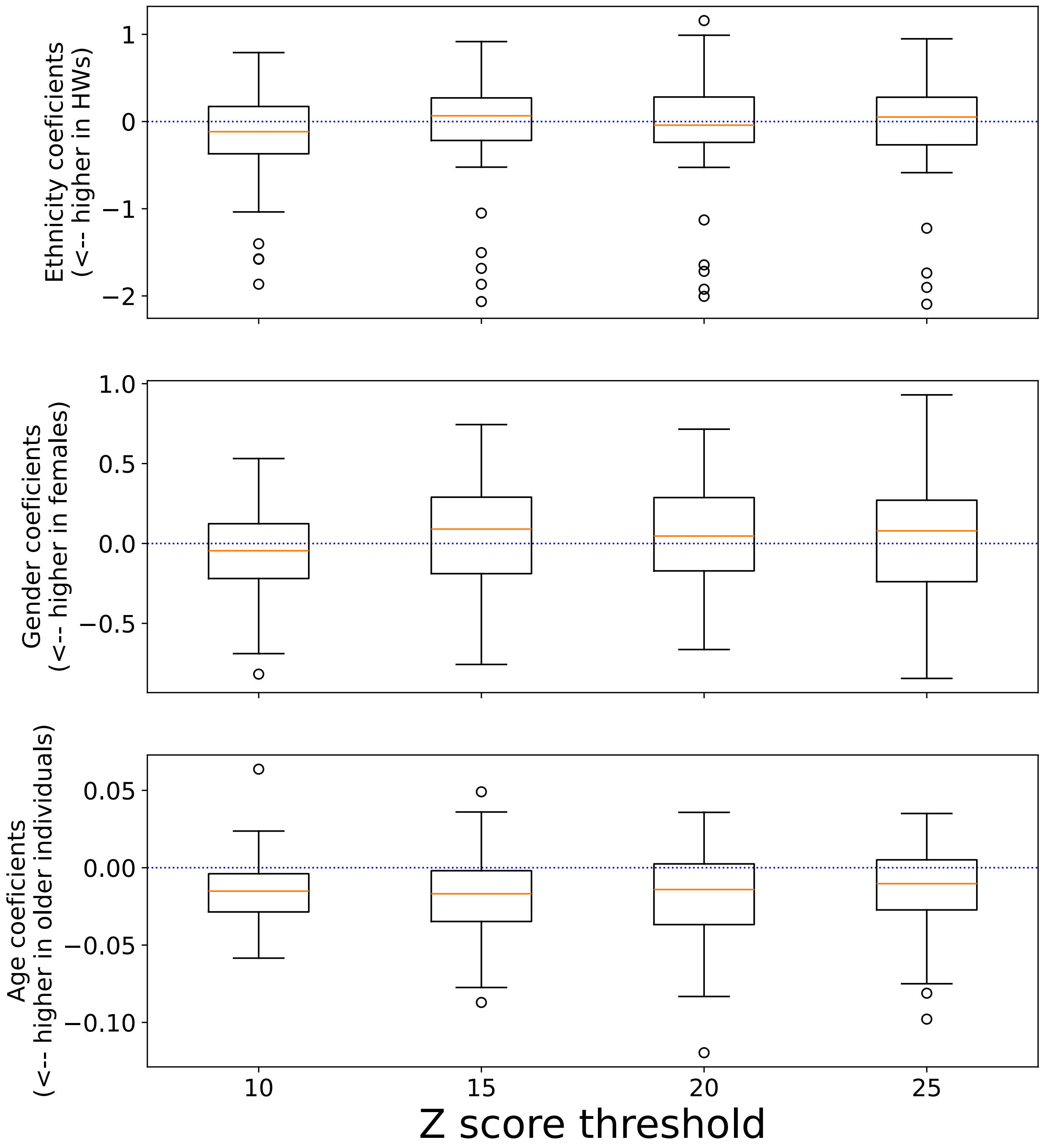

Supplemental Figure 4. Trends in GLM variable coefficients across all Z score thresholds. Box plot summary of overall patterns in coefficient distribution for each independent variable included in the GLM analysis. The orange line within each box represents the median, while the lower and upper bounds of each box represent the 1st and 3rd quartiles, respectively. The whiskers extend to points that lie within 1.5 interquartile ranges of the 1st and 3rd quartiles. Values that fall outside of the boundaries of the whiskers are plotted as individual circles.

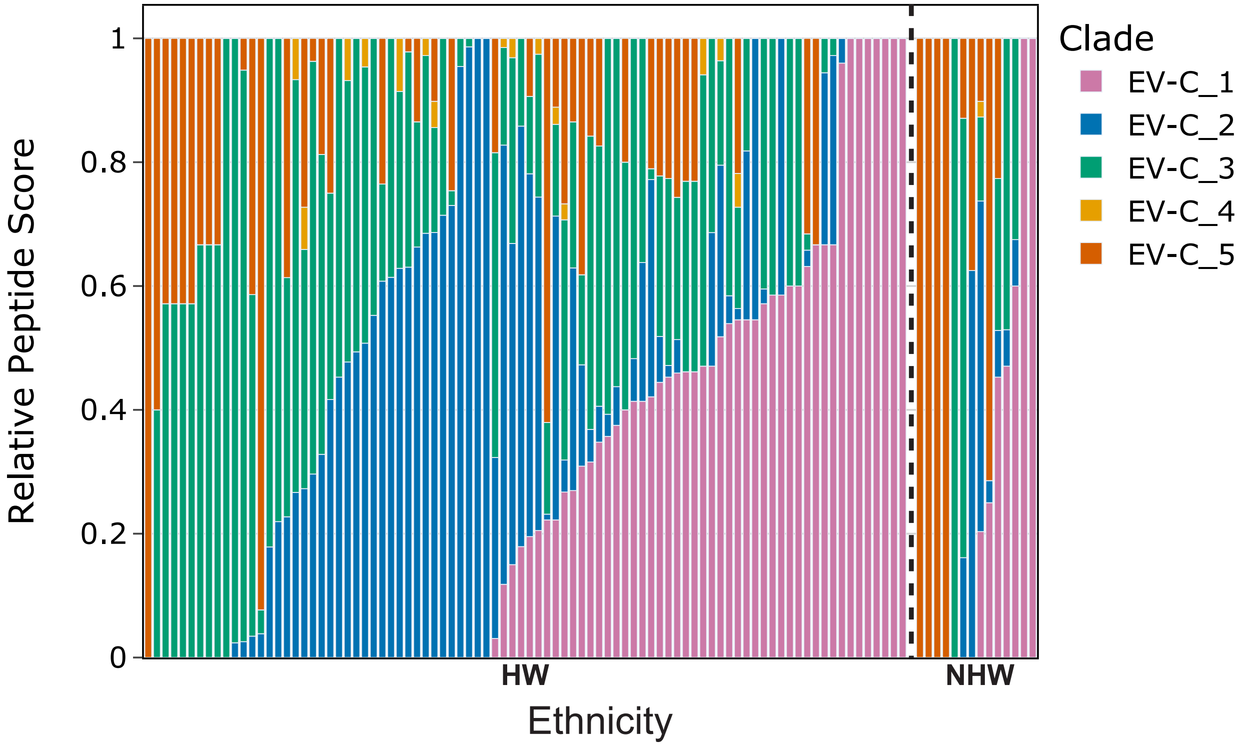

Supplemental Figure 5. Individual level antibody reactivity against clade-specific "Other EV-C" peptides. Bar plot showing clade-specific relative peptide scores for the subset of enriched peptides in all HW and NHW individuals predicted to be seropositive against "Other EV-C" (Figure 6B) and with a total clade-specific score greater than 20. Each bar represents an individual donor. Each color represents one of the "Other EV-C" phylogenetic clades shown in Figure 7A.

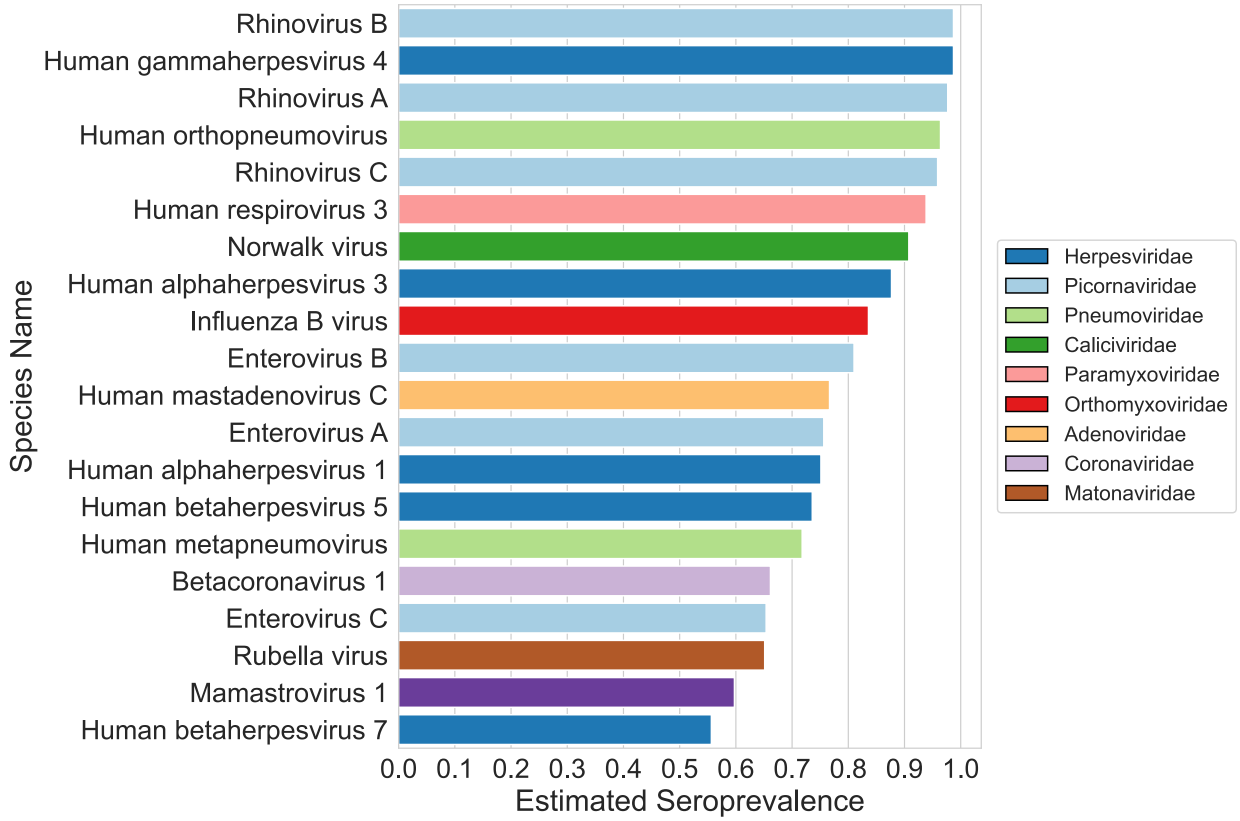

Supplemental Figure 6. The most common human viruses in our cohort based on PepSeq estimates of seroprevalence. Estimated seroprevalence is shown for the 20 most prevalent virus species across our entire patient cohort (n=390) using a Z score threshold of 15 and our representation normalized seropositivity score thresholds (Table S6). Bar colors indicate viral family. Full seroprevalence data can be found in Table S7.
